# Analysis of clinical effect of comprehensive nursing on patients with slow transit constipation after laparoscopic surgery

**DOI:** 10.4314/ahs.v25i3.24

**Published:** 2025-09

**Authors:** Panpan Wang, Yan Zhou, Ying Wang, Pengtao Ren, Lin Lin, Huan Ren

**Affiliations:** 1 Second Hospital of Hebei Medical University, Hebei, Shijiazhuang, 050000; 2 The First Hospital of Hebei Medical University, Hebei, Shijiazhuang, 050031

**Keywords:** Anxiety, Comprehensive nursing, Depression, Quality of life, Satisfaction, Slow transit constipation laparoscopy

## Abstract

**Objective:**

To explore the clinical effect of comprehensive nursing on patients with slow transit constipation after laparoscopic surgery.

**Methods:**

A total of 80 patients with colonic slow transit constipation, who received laparoscopic treatment in our hospital from January 2019 to December 2021 were selected. A total of 80 patients with colonic slow transit constipation treated by laparoscopy were divided according to the random table method. There were 2 groups, 40 cases in the control group and 40 cases in the experimental group. All patients underwent laparoscopic surgery, the control group was given routine nursing measures, and the experimental group was given comprehensive nursing intervention on the basis of routine nursing. The SAS and SDS scores were used to compare the levels of anxiety and depression in the two groups before and after the intervention; the quality of life core scale (QOL-C30) was used to score the physical function, material function, psychological function and social function, and the two groups of patients were compared before and after the intervention. The quality of life; comparison of postoperative pain scores between the two groups; comparison of nursing satisfaction between the two groups were analyzed.

**Results:**

SDS and SAS scores of the control group before the intervention and the experimental group before the intervention (P<0.05). Compared with the control group after the intervention, the SDS and SAS scores of the experimental group after the intervention were significantly lower (P<0.05). There was no significant difference in the quality of life between the two groups before the intervention (P>0.05); compared with before and after the intervention, the quality of life in the two groups after the intervention was significantly higher than that before the intervention (P<0.05); compared with the control group after the intervention The quality of life of patients in the experimental group after intervention was significantly improved (P<0.05). The satisfaction rate of the patients in the control group was 62.5%; the satisfaction rate of the patients in the research group was 92.5%; compared with the control group, the satisfaction rate of the patients in the research group was significantly higher (P<0.05).

**Conclusion:**

Comprehensive nursing can effectively improve the negative emotions of patients with colonic slow transit constipation after laparoscopic surgery, improve the quality of life and satisfaction of patients, and reduce postoperative pain.

## Introduction

Colonic slow transit constipation, one of the common clinical diseases, is a refractory defecation disorder characterized by the prolonged passage of food through the gastrointestinal tract, and the weakened colonic motility, without organic lesions found in the colon^1^-^2^. Colonic slow transit constipation generally affects women of childbearing age, with increasing incidence in recent years. Its clinical symptoms mainly include decreased frequency of bowel movements, less stool or even disappearance of stool intention, and hard stool with abdominal distension^3^-^4^. Its etiology may be closely related to bad defecation habits, abnormal diet, disorder of mental endocrine, and abnormal secretion of gastrointestinal regulatory peptide. The symptoms were stubborn, and will be worsen with the passage of time, causing a serious impact on psychology and quality of life of the patients^5^-^6^. At present, surgery is still an effective method for the treatment of colonic slow transit constipation. In recent years, with the development of laparoscopy, laparoscopic subtotal colectomy and total colectomy have been widely used for the treatment of patients with severe colonic slow transit constipation after invalid conservative treatment. By virtue of the advantages such as small trauma, quick recovery, and better protection of pelvic autonomic nerves, laparoscopy has been widely used in clinical practice, with a definite therapeutic effect^7^-^8^. However, a study^9^ found that long-term constipation in patients with colonic slow transit constipation would lead to stress incontinence, and unhealthy emotions such as anxiety and depression, which would seriously affect the quality of life and prognosis of the patients, thus hindering the improvement of their quality of life. The rationality and normalization of nursing intervention is quite iportant for the postoperative quality of life. Traditional nursing is limited to basic nursing, with a lagging concept of nursing^10^-^11^. As a new and targeted intervention mode, comprehensive nursing can take targeted nursing measures according to the conditions of each patient, to meet the requirements of disease conditions for nursing. In addition, comprehensive nursing, with a satisfactory nursing effect, has been widely used in clinical treatment^12^. In this study, comprehensive nursing was performed for the patients with colonic slow transit constipation after laparoscopic surgery, with the aim of observing its effect on clinical effect.

## Materials and methods

### General data

A total of 80 patients with colonic slow transit constipation, who received laparoscopic treatment in Second Hospital of Hebei Medical University, Hebei, Shijiazhuang hospital from January 2019 to December 2021 were selected. A total of 80 patients with colonic slow transit constipation treated by laparoscopy were divided into 2 groups according to the random table method, 40 cases in the control group and 40 cases in the experimental group. In the control group, 18 male and 22 female patients at the age of 25-55 were included, with the average age of 35.46±3.24 years old. There were 16 moderate patients and 24 severe patients. As for the educational level, the quantity of the patients with the educational levels of junior high school, senior high school and junior college, and bachelor above were 5, 15 and 20 respectively. In the experimental group, 19 male and 21 female patients at the age of 28-58 were included, with the average age of (36.11±3.08) years old. There were 18 moderate patients and 22 severe patients. As for the educational level, the quantity of the patients with the educational levels of junior high school, senior high school and junior college, and bachelor or above was 6, 16 and 18 respectively. There was no significant difference in gender, age, severity of disease and educational level between the two groups (P>0.05).

### Inclusion and exclusion criteria

Inclusion criteria: (1)Patients with colonic slow transit constipation diagnosed by abdominal CT, B-ultrasonography, and microscopy in combination with medical history and physical signs; (2)Patients with time of onset of more than 1 year, who should be treated by operation due to invalid drug therapy; (3) Patients with a strong desire for surgery and surgical indications. Exclusion criteria:(1) Patients failing to actively participate in this study; (2)Patients with mental illness in the past; (3)Patients with communication disorders.

### Surgical method

All patients underwent laparoscopic surgery. The patients without colonic tension underwent laparoscopic total colectomy, and ileorectostomy; and those with without colonic tension in local positions underwent subtotal colectomy and caeco-rectal anastomosis.

### Nursing measures

The control group was given routine nursing measures, including preoperative bowel preparation, health education/contraindication guidance, and postoperative routine nursing. The experimental group was given comprehensive nursing intervention on the basis of routine nursing, including: (1) Psychological intervention: The charge nurse should communicate with the patient in a timely manner after admission, to fully understand the patient's conditions, observe the key symptoms and treatment points, diet and nutritional status, and self-care ability. Prior to the operation, the charge nurse should focus on the subjective symptoms of the patient, especially his/her mental and psychological symptoms, and timely inform the patient of the knowledge about the conditions and operation, as well as the matters needing attention during the operation. The nurse should also introduce the successful surgical cases to increase the patient's confidence, and also establish a good relationship with the patient, to provide more individualized and humanized services. (2) Dietary guidance: The nurse should prepare a healthy diet plan for the patient after the operation, notify the dietary precautions, and ask the patient to take more soluble dietary fiber and water, and do more exercises appropriately, which can promote intestinal peristalsis and help to treat constipation. (3) Exercise guidance and abdominal massage: The nurse should guide the patient to perform levator ani movement, so as to enable the patient to achieve spontaneous contraction and relaxation after fully feeling the contraction and relaxation of sphincter. The nurse should also provide appropriate abdominal massage. On each morning after waking up and at the time before sleeping, the nurse should ask the patient to empty the bladder, lie supine with bent knees, and relax the abdomen. The nurse should put hands on the right lower quadrant of abdomen to massage clockwise, with the strength of sinking for 1cm, which could be increased for the massage of the lower left abdomen before feeling thpain. The massage can be stopped when the skin becomes warm, and during the massage, the nurse should ask the patient's feeling and whether he/she is uncomfortable. (4) Defecation guidance: The nurse should provide individualized instruction for different patients, analyze the specific causes of constipation, and help the patient to form a correct defecation habit. Since colonic motility is the strongest after waking up in the morning, the medical staff should encourage the patient to defecate after getting up, or after meals based on gastrocolic reflex. (5) Relieving of postoperative pain: After the operation, the medical staff can chat with the patient and play music, to shift the attention to postoperative pain. (6) Prevention and treatment of postoperative complications: Before the operation, the medical staff should tell the patient about the postoperative complications, and provide enough time to get psychologically prepared. The medical staff may determine the solutions based on the possible complications. The patients in both groups should be continuously intervened for 4 weeks.

### Evaluation indicators

Selfrating Anxiety Scale (SAS) was used to evaluate anxiety of each patient. The cut-off value of standard SAS score is 50. Mild anxiety: 50-59, moderate anxiety: 60-69, severe anxiety: above 70. Self-rating Depression Scale (SDS) was used to evaluate the depression of each patient. The cut-off value of standard SDS score is 53. Mild depression: 53-62, moderate depression: 63-72, severe depression: above 73. The Quality of Life Core Scale (QOL-C30) was used to evaluate the quality of life of patients in the two groups, involving physical function, material function, psychological function and social function before and after intervention. The quality of life score is 100 in total, and the higher score indicates better quality of life. Evaluation of postoperative pain: The Numeric Rating Scale (NRS) consisting of 10 points was used to evaluate the postoperative pain: Mild pain: 0-3, which would not affect sleep, moderate but tolerable pain: 4-6, which would slightly affect sleep, severe pain: 7-10, which would seriously affect sleep. Nursing satisfaction: Questionnaires were used to evaluate the nursing satisfaction among the patients. It adopts a 100-score system: High satisfaction: 80-100, general satisfaction: 60-79, dissatisfaction: 0-59.

### Statistical analysis

The data were analyzed by Graphpad priam 8.0 software, and the measurement data were expressed as *x̅±s*. t test was performed for those conforming to the normal distribution, and Wilcoxon rank-sum test was performed for those not conforming to the normal distribution. The measurement data were tested by χ2, and P<0.05 indicated that the difference was statistically significant.

## Results

### Comparison of SDS scores between the two groups of patients

There was no significant difference in SDS score between the control group and experimental group before the intervention (P<0.05). The comparison before and after the intervention indicated that SDS score of the two groups of patients after the intervention was lower than before the intervention (P<0.05). Compared with the control group after the intervention, SDS score of the patients in the experimental group after the intervention significantly decreased (P<0.05) (Table 1).

**Table 1:** Comparison of SDS score between the two groups of patients (*x̅±s*)

		SDS	
Group	n	Before the intervention	After the intervention
Control group	40	67.42±2.18	56.92±1.95*
Experimental group	40	67.83±2.25	42.34±1.83*^#^
*t*		0.8277	34.48
*P*		2.4104	<0.0001

**Note:** Compared with the situation before the intervention

* P<0.05; compared with the control group

# P<0.05

### Comparison of quality of SAS scores of the two groups of patients

There was no significant difference in SAS score between the control group and experimental group before the intervention (P>0.05). Compared with the scores before the intervention, SAS score of the two groups of patients significantly decreased after the intervention (P<0.05). Compared with the control group after the intervention, SAS score of the patients in the experimental group after the intervention significantly decreased (P<0.05) (Table 2).

**Table 2:** Comparison of SAS score between the two groups of patients (x̅±s)

		SAS	
Group	n	Before the intervention	After the intervention
Control group	40	64.78±2.31	53.28±1.57*
Experimental group	40	65.01±2.45	43.17±1.32*^#^
*t*		0.4320	31.17
*P*		0.6669	<0.000

**Note:** Compared with the situation before the intervention

* P<0.05; compared with the control group

# P<0.05

### Comparison of quality of life of the two groups of patients

There was no significant difference in quality of life of the two groups of patients before the intervention (P>0.05). Compared with before and after the intervention, the quality of life of the two groups of patients after the intervention was significantly higher than that before the intervention (P<0.05). Compared with the control group after the intervention, the quality of life of patients in the experimental group after the intervention significantly increased (P<0.05) (Table 3).

**Table 3:** Comparison of quality of life between the two groups of patients (x̅±s)

Group	n	Before the intervention	After the intervention
Control group	40	51.13±2.25	66.37±2.49*
Experimental group	40	51.48±2.31	81.64±3.11*^#^
*t*		0.6865	24.24
*P*		0.4945	<0.0001

**Note:** Compared with the situation before the intervention

* P<0.05; compared with the control group

# P<0.05

### Comparison of postoperative pain score between the two groups of patients

Compared with the control group, the postoperative pain score of patients in the experimental group significantly decreased (P<0.05) (Table 4).

**Table 4:** Comparison of postoperative pain score between the two groups of patients (x̅±s)

Group	n	NRS
Control group	40	5.26±1.03
Experimental group	40	2.47±0.86*
*t*		13.15
*P*		<0.0001

Note: Compared with the control group

* P<0.05

### Comparison of degree of satisfaction between the two groups of patients

The degree of satisfaction in the control group was 62.5%, which was 92.5% in the experimental group. Compared with the control group, degree of satisfaction in the experimental group increased significantly (P<0.05) (Table 5).

**Table 5:** Comparison of degree of satisfaction between the two groups of patients (%)

Group	n	High satisfaction	General satisfaction	Dissatisfaction	Degree of satisfaction %
Control group	40	10	15	15	25 (62.5)
Experiment al group	40	18	19	3	37 (92.5)
*χ^2^*					26.59
*P*					<0.0001

## Discussion

Colonic slow transit constipation, known as colonic inertia, is an intestinal motility disorder. Due to the long course and unclear etiology of colonic slow transit constipation, and the insufficient attention to the disease at the early stage, the treatment effect was not ideal, which seriously affected the quality of life of the patients^13^-^14^. In clinical practice, conservative methods such as diet therapy, muscle strength training, defecation training and drug therapy are generally used to treat the patients with colonic slow transit constipation^15^-^16^. Laparoscopic surgery is a method for treating the patients after failure of conservative treatment. The treatment of colonic slow transit constipation with laparoscopic subtotal colectomy can relieve the symptoms and improve the quality of life of the patients in an ideal manner^17^. Comprehensive nursing in the perioperative period, including psychological and physiological nursing, can reduce the psychological burden of the patients, and make them aware of the disease and operation. Therefore, the patients can actively and effectively assist in the operation and treatment, thus promoting the prognosis of the patients. In a study of Ye and Chen^18^, colonic slow transit constipation was found to be a biological, psychological and social disease with multiple epidemic factors, including diet and psychological factors. In addition, the patients would be affected by anxiety and depression, which would increase the risk factor of the operation.

Therefore, it is particularly important to provide effective nursing for the patients before and after the operation. With the development and transformation of medical modes, great changes have taken place in nursing mode. The improvement of nursing based on the strengthening of basic nursing has become a hot topic in clinical research currently. Comprehensive service is a new and comprehensive service mode, and its connotation is to meet the basic living needs of the patients, and ensure the safety and comfort of nursing. It can help the patients to adjust the mental state and maintain balance. The medical staff should try to get the coordination and support from the patients and their family members, and the social system, so as to provide the patients with personalized nursing, thus improving the patients' satisfaction. Comprehensive nursing mode can achieve the comprehensive and individualized patient-centered nursing in a real sense. As found in a study of Tao et al.^19^, almost all the patients with colonic slow transit constipation had different degrees of negative emotions such as anxiety and depression, and in addition to the prolonging of the disease due to the conservative treatment, the difficulty of treatment increased, with higher medical expenses. These conditions caused great pain and psychological disorder to the patients, seriously deteriorating the quality of life of the patients. The results of this study indicated that SDS and SAS scores of the patients in the experimental group after the intervention were significantly lower than those in the control group. Compared between the two groups after the intervention, SDS and SAS scores of the patients in the experimental group were significantly lower than those in the control group.^19^ found that high-quality nursing can significantly improve the anxiety and depression of the patients with colonic slow transit constipation and their quality of life. In addition, it can also increase the nursing satisfaction of the patients. The results of this study showed that the quality of life and patient satisfaction of the patients with colonic slow transit constipation underwent comprehensive nursing were also significantly higher than those in the control group, which confirmed the study results of Tao et al.^19^. Pain is a painful emotional experience and subjective feeling.

The result of pain evaluation is not the self-judgment of the patients' feeling by the medical staff, but the reflection of the patients' subjective feelings. The results of this study showed that the postoperative pain score of the patients in the experimental group after comprehensive nursing was significantly lower than that in the control group, suggesting that comprehensive nursing can relieve the pain of colonic slow transit constipation.^20^ confirmed that comprehensive nursing could effectively relieve the clinical symptoms of the patients with slow transit constipation, promote effective defecation of the patients, and improve their quality of life. In conclusion, comprehensive nursing can effectively improve the negative emotions of patients with colonic slow transit constipation after laparoscopic surgery, improve the quality of life and satisfaction of patients, and reduce postoperative pain.

## Data Availability

The data and materials of this experiment are available.

## Project Source

Hebei Province 2025 Annual Medical Science Research Project (No.: 20250410).
